# Hepatitis E virus (HEV)—The Future

**DOI:** 10.3390/v11030251

**Published:** 2019-03-13

**Authors:** Joachim Denner

**Affiliations:** Robert Koch Institute, 13353 Berlin, Germany; DennerJ@rki.de; Tel.: +49-30-18754-2800

**Keywords:** hepatitis E virus, hepatitis, blood donations, elimination programs, emerging diseases, one health

## Abstract

Hepatitis (HEV) is widely distributed in pigs and is transmitted with increasing numbers to humans by contact with pigs, contaminated food and blood transfusion. The virus is mostly apathogenic in pigs but may enhance the pathogenicity of other pig viruses. In humans, infection can lead to acute and chronic hepatitis and extrahepatic manifestations. In order to stop the emerging infection, effective counter-measures are required. First of all, transmission by blood products can be prevented by screening all blood donations. Meat and sausages should be appropriately cooked. Elimination of the virus from the entire pork production can be achieved by sensitive testing and elimination programs including early weaning, colostrum deprivation, Caesarean delivery, embryo transfer, treatment with antivirals, protection from de novo infection, and possibly vaccination. In addition, contaminated water, shellfish, vegetables, and fruits by HEV-contaminated manure should be avoided. A special situation is given in xenotransplantation using pig cells, tissues or organs in order to alleviate the lack of human transplants. The elimination of HEV from pigs, other animals and humans is consistent with the One Health concept, preventing subclinical infections in the animals as well as preventing transmission to humans and disease.

## 1. Introduction

Hepatitis E virus (HEV) is a single-stranded RNA virus approximately 7.2 kb in length with three open reading frames (ORFs). It is non-enveloped in bile and feces and is present coated in a lipid membrane (quasi-enveloped) in blood. HEV belongs to the genus *Orthohepevirus* in the *Hepeviridae* family. This genus includes four species, *Orthohepesvirus A* with eight genotypes, the *Orthohepesvirus B* circulating in chicken, the *Orthohepesvirus* C in rats and ferrets, and the *Orthohepesvirus D* in bats [1]. Five members of the *Orthohepesvirus A* are found to infect humans [2]. HEV genotype 1 (HEV-1) and HEV-2 are human viruses. They are highly endemic in several parts of Asia, Africa, the Middle East, and Mexico. They are spread through contamination of water supplies with human feces [3]. Every year, HEV-1 and HEV-2 cause 20 million new infections, 3.4 million acute hepatitis E, and 70,000 deaths from acute liver failure [4]. The seroprevalence rate of anti-HEV antibodies in most parts of Africa and Asia is 10–40%, in Egypt around 80% [3]. In contrast, HEV-3 and HEV-4 are zoonotic viruses that are able to infect humans, pigs and other animal species. Transspecies transmission occurs by direct contact with infected animals, and consumption of HEV-contaminated food products [5,6,7,8,9,10]. Transmission of HEV from infected humans to other humans by blood transfusion [11,12,13,14,15,16,17,18,19,20,21,22,23,24,25] and organ transplantation [26,27,28,29] has also been observed. HEV-1, HEV-2, HEV-3, and HEV-4 cause self-limiting acute hepatitis, acute liver failure, and neurological illness, however, different pathologies are associated with different strains. Genotypes 3 and 4 are by far more associated with neurological conditions than genotypes 1 and 2. Whereas, high mortality rates in pregnant woman and pancreatitis are more associated with genotypes 1 and 2 [30,31]. Pre-existing liver diseases and age are additional risk factors [32]. HEV-3 infections may lead to acute-on-chronic liver failure in patients with underlying liver disease. Immunosuppressed individuals are at risk for developing chronic infections which may lead to liver fibrosis and cirrhosis. The prevalence of hepatitis E in developed, industrialized countries differs between regions. The seroprevalence of hepatitis E in the southeast of France reaches over 50%. In recent years, approximately 68,000 HEV infections were counted in France, 100,000 in the United Kingdom and 300,000 in Germany per year based on HEV-specific antibodies [3,33]. The number of infections is increasing dramatically, partially due to more frequent testing and better detection methods [34]. Genotypes 5 and 6 have been found in wild boars in Japan [35]. Infection with HEV-7 was observed in dromedary camels [36] and HEV-8 was found in Bactrian camels [37]. 

## 2. HEV-3 in the Pig Population and Other Wild Animals

HEV-3 is widely distributed in the pigs from the Americas, Europe, Africa, Japan, South-East Asia, and Oceania, whereas HEV-4 was found mainly in pigs from China, Japan, and Indonesia. The seroprevalences were estimated between 5% and 100% [38]. HEV-4 was also found in European countries [39]. The prevalence of the virus depended on the age of the animal, the material tested, and the method used for testing (PCR based or immunological). Usually, infection was found at an early age after loss of the maternal antibodies. Viral secretion was detected 3 to 8 weeks after weaning. The main site of HEV replication is the liver, but the virus was also found in other organs, primarily in the small intestines, lymph nodes, and colons [40]. The virus is mainly excreted fecally leading to an oro-fecal transmission. The virus load was high in all types of herds (weaners, growers, and fatteners), but is found to be the highest in fatteners. The seroprevalence was slightly higher in organic farms compared with conventional and free-range farms [41]. Infection with HEV alone has little impact on pig health, and no clinical symptoms were detected. However, it is important to note that HEV infection may enhance the disease caused by other porcine viruses. In one case co-infection with the immunomodulatory porcine reproductive and respiratory syndrome virus (PRRSV) caused fatal disease in piglets [42]. In another case, co-infection with the porcine circovirus 2 (PCV2) exacerbated the HEV release [43]. Hints that the immune modulatory effects of PCV2 combined with HEV influence the disease pattern in pigs was also found in other cases [44]. More evidence is required to affirm that HEV infection enhances the disease caused by other porcine viruses. The reason for the enhancing activity may lay in the immunosuppressive properties of HEV. When immunocompromised pigs were infected with HEV, serum levels of Th1 cytokines (IL-2 and IL-12) were reduced, whereas, importantly, the immunosuppressive drug treatment alone without HEV infection did not suppress these cytokines [45]. This finding indicates that HEV infection actively suppresses Th1 immune responses in immunocompromised pigs, as has been observed in human patients that are chronically infected with HEV [31].

HEV-3 and HEV-4 were also detected in wild boars, in most cases without clinical symptoms. However, HEV circulates at lower rates in wild boars than in domestic pigs (for review see [38]). An unexpected finding was that deer could get infected in the wild with HEV. In Germany, HEV was found in three deer species, i.e., red, roe, and fallow deer [46,47]. Anti-HEV-antibodies and HEV RNA were found in red, roe, sika, fallow and white-tailed deer in America, Asia, and Europe (for review see [38,48]), where all HEV sequences belonged to HEV-3. However, lower virus loads were found in livers from deer compared with livers from wild boars [47].

HEV was also found in other species in addition to pigs and deer. Ruminants and others species have shown to be susceptible to HEV (for review see [48]), the same was observed in rabbits [48,49]. When HEV infection was analyzed in equines in Spain, HEV RNA in serum was detected in 0.4% of the horses, 1.2% of the donkeys and 3.6% of the mules [50].

While contaminated water is the main cause of transmitting HEV-1 and HEV-2, zoonotic virus distribution via water has also been observed [51].

## 3. Transmission of HEV to Humans

Reports from many European countries, America, Africa, and Asia, show the detection of HEV in the liver, meat and meat products, mainly sausages with and without liver (for review see [38]). The detection rate ranged from 3 to 38% of all tested samples. HEV genome concentrations between 20 and 10^7^ RNA copies/g were found. Since there is no efficient cell culture system for the detection of infectious virus, it was difficult to analyze whether the virus is still infectious. However, in a few cell systems, infectivity of food samples was shown [52,53]. In addition, inoculation of sample homogenates into pigs also demonstrated the presence of infectious virus in commercially sold liver [54]. The final evidence that the virus in the meat is infectious came from reports of infections occurring in humans after eating undercooked meat or sausages [5,6]. Identical HEV sequences have been found in the meat and infected individuals [10]. HEV transmission was also linked to the consumption of meat from sika deer in Japan, where identical HEV sequences were identified in the patients and the meat [55].

Infection with HEV was also observed after direct contact exposure. Numerous studies demonstrated a higher seroprevalence in individuals who had contact with pigs (for review see [38]). Transmission of HEV was also observed in forest workers and hunters [16,17]. In rare cases, evidence of induction of disease after transmission of HEV by contact such as frequent contact to a pet pig, surgeon training, and slaughterhouse work was also obtained (for review see [38]).

It was shown that many blood donors were infected with HEV and that HEV has been transmitted from human to human by blood transfusion [11,12,13,14,15,16,17,18,19,20,21,22,23,24,25]. Among blood donors, 16% were found seropositive in the United States and Southwest England, 21% in Denmark, 52% in the southeast of France, between 7% and 30% in Germany, and 29.4% in Switzerland. Transmission of HEV by blood transfusion can be dangerous for the recipients, considering their immunsuppressive status, underlying disease or other circumstances requiring a blood transfusion. HEV transmission was also associated with organ transplantation [26,27,28]. The prevalence of persistent HEV infection in patients with solid organ transplantation in Western Europe varies between 0.7% [29] and 5.3% [28].

HEV was also transmitted from ruminants and rabbits to humans. Cattle farmers in Lao, for example, had a higher risk of HEV infection than other villagers [56] and a high seroprevalence of hepatitis E virus was observed in rabbit slaughterhouse workers [57].

HEV-7 was also transmitted to a human, a liver transplant recipient who regularly consumed camel meat and milk [36].

## 4. Measures to Prevent HEV Transmission

Transmission of HEV by blood transfusion may be reduced by testing the blood donations. Several arguments are in favor of testing all blood donations for HEV-3 to prevent transmission [58]. These arguments include: (i) Testing only the donations for people at risk may be less expensive. However, these patients are difficult to define. People at risk are not only immunosuppressed patients such as transplant recipients or patients with HIV infection but also include cancer patients under chemotherapy or patients with rheumatic diseases. (ii) Clinical effects of an HEV infections had also been observed in immunocompetent individuals with different underlying diseases such as thrombotic thrombocytopenic purpura [59] or systemic lupus erythematosus [60]. (iii) The handling of two different types of donations may be difficult and expensive, and (iv) It is nearly impossible for a physician to assign a patient to an HEV risk in event of an emergency that requires blood transfusion [58]. A nationwide HEV RNA universal screening of blood donations has already been introduced in some industrialized countries such as Ireland, the UK, Japan, the Netherlands and recently, Germany. Blood authorities in Greece, Portugal, Italy, France, and Spain are evaluating the situation [61]. Detection of HEV by PCR-based methods will identify blood donors with detectable viral RNA genome (above the detection limit). Using immunological methods, more antibody-positive donors compared with RNA-positive donors can be detected. However, until now no viral transmission from HEV antibody-positive HEV RNA negative (below the detection limit) donors has been reported. Furthermore, not all PCR-positive blood donations caused an infection and a good correlation between virus load, and transmissibility was observed [11,13,23]. 

It is clear from the evidence that HEV-contaminated food is the primary source of HEV transmission. To prevent transmission by food, appropriate treatment of the food is required to inactivate the virus. This includes testing of highly contaminated organs and their elimination as well as heat treatment to inactivate the virus which is common in undercooked meat and sausages [5,6,10]. Safety measures should also be undertaken in the case of HEV-contaminated shellfish [62,63], vegetables, and fruits [64], which are possibly contaminated by pig manure. These measures may include appropriate washing during processing on the one side, and prohibition of the use of manure from HEV-infected animals in the field on the other side. Alternatively, the manure could also be inactivated. These steps, in addition to heat treatment of water, will also reduce the risk of HEV transmission by water.

**Figure 1:**
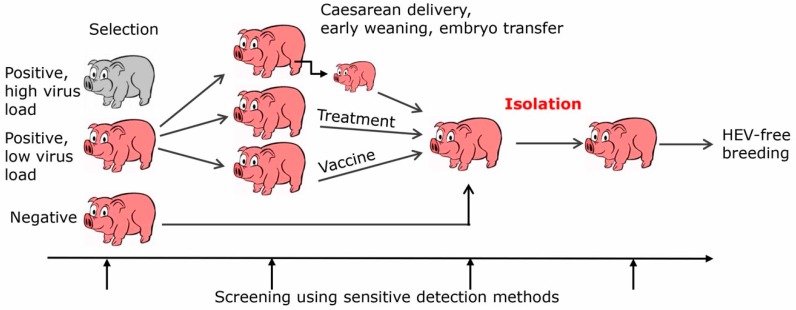
Schematic presentation of strategies to eliminate HEV from pig herds generated for xenotransplantation or pork production.

A more efficient but difficult to achieve way to prevent transmission of HEV from pigs to humans is by eliminating HEV entirely from the pork production (Figure 1). There are several ways to achieve this eradication, firstly by selection and breeding of HEV-negative animals, secondly by early weaning, Caesarean delivery, colostrum deprivation, embryo transfer, and finally by vaccination or treatment with an antiviral. Since HEV can be transmitted by milk and colostrum, early weaning, colostrum deprivation and Caesarean delivery may reduce the transmission. However, it must be considered that HEV can be transmitted via the placenta [65]. In this case, embryo transfer may be the only way to prevent transmission. Ribavirin is one of the effective antivirals, it is a nucleoside inhibitor used to stop viral RNA synthesis. It has been successfully applied to treat patients infected with HEV [66,67]. Only a minority of chronically HEV infected patients fail to achieve sustained virological response, possibly because of viral mutants [66]. Although there are no reports showing treatment of pigs with ribavirin, adverse effects and pharmacokinetics of the drug have been studied in pigs [68] and it has been used to treat other virus infections in pigs, e.g., porcine nidovirus [69]. There is no need to treat all animals of the herd, it may be more effective to start a new herd with a few ribavirin-treated HEV-negative animals. Furthermore, other antiviral substances are in the pipeline, for example zinc salts [70] or sofosbuvir, which is registered for the treatment of hepatitis C [71]. 

Effective vaccines are always the best protection from a virus infection. Therefore, HEV vaccination is urgently needed. A vaccine based on a protein encoded by open reading frame 2 (ORF2) of HEV-1 has been successfully applied in China in a large human population with high efficiency [72,73,74]. Most importantly, research substantiated that the vaccine cross protects against the HEV-4 in human beings [75]. Obviously, all genotypes represent one single serotype [76].

When eliminating HEV from a production facility, stringent hygienic conditions should be applied, and the animals should be kept in appropriate isolation to avoid de novo infection by HEV. Furthermore, infection of pigs by human handlers has to be prevented. In the case of free range farms, animals should be protected from infection by wild boars, e.g., by a solid wall. Possibly, in far future HEV can be controlled and eliminated from the wild animals using oral vaccination as was successfully done in oral rabies vaccination campaigns for foxes and raccoon dogs [77]. In addition to wild boars, other wild animals can be HEV-positive and can transmit the virus. Most interestingly, recently a rat with HEV belonging to the species *Orthohepevirus C* was linked to severly acute hepatitis in an immunocompetent patient [78], and an HEV infection in a liver transplant patient [79]. Although transmission of an *Orthohepevirus C* from rats to pigs seems unlikely, pigsties should be built in a way to avoid contact with rats. It is important to note that due to the extremely high import and export rates, elimination of HEV from pigs production has to be an international effort.

Starting with a small number of HEV-negative animals, pig production in farms can be organized free of HEV, and when these farms are certified as HEV-free, testing of all animals is no longer required, reducing the costs significantly. Based on such certification the consumer may decide whether he prefers meat from a HEV-free certified slaughterhouse (which may be more expensive) or not. A close control should be organized and certifications should be withdrawn in the case of violation. A similar certification should be given to fruits from fields that are not treated with manure from HEV-positive animals.

Eradication of HEV in pigs would significantly prevent its transmission to humans, prevent HEV-induced liver diseases, prevent chronic infection in immunocompromised individuals, and prevent subclinical infections. The impact of these subclinical infections in humans is still unknown. In pigs, where HEV is also non-pathogenic, co-infection of the animals with HEV and other pathogenic pig viruses leading to an enhancement of the disease was observed [42,43]. It may be because such an enhancement can also be expected in the case of co-infections of HEV with other human viruses. For example, exacerbated clinical manifestations are found after co-infections of patients with Hepatitis A virus and HEV-1 [80].

Whilst pigs are the main source of zoonotic HEV infection, the virus is also found in deer, rabbits, cattle, and dromedaris [38,45,46,47,48,50]. In order to eliminate the problem of zoonotic infection for humans, these sources of infection must be eliminated too. Having this in mind, it is rather difficult to evaluate the economic cost-benefit balance of HEV-3 eradication and whether it will be possible to prevent diseases and subclinical infections. Furthermore, it will be difficult to decide whether testing for HEV of all blood donations will no longer be required. 

## 5. HEV and Xenotransplantation

Xenotransplantation using pig cells, tissues, and organs was developed to alleviate the increasing shortage of human transplants for the treatment of tissue and organ failure. Using multiple genetically modified animals and new immunosuppressive regimens, a considerable success with long survival times in pig to non-human primate xenotransplantations has been achieved [81,82]. For example, 195 days in the case of orthotopic heart transplantations into baboons [83] and 603 days in the case of islet cell transplantation [84]. Xenotransplantation may be associated with the risk of transmission of porcine microorganisms including viruses to the transplant recipient. HEV is one of the potentially zoonotic viruses [85] and it was found in triple genetically modified (Denner, J.; Robert Koch Institute, Berlin, Germany, 2018) and non-modified animals [64] produced and bred for xenotransplantation. However, until now no HEV has been transmitted to non-human primates in preclinical trials [86]. In the first clinical trials using islet cells from Auckland Island pig for the treatment of diabetes, no HEV was transmitted because Auckland Island pigs were free from HEV [87,88,89]. Therefore, in order to prevent HEV transmission, elimination strategies as shown in Figure 1 should be applied [85]. The elimination strategies could first be introduced in pig breeds used for xenotransplantation and if successful, then used for the pork industry.

## 6. HEV and the One Health Concept

The One Health concept recognizes that the health of people is connected to the health of animals and the environment. The elimination of HEV from pigs, other animals, and humans is consistent with the One Health concept, preventing subclinical infections in animals as well as preventing transmission and disease in humans. In this context, xenotransplantation holds a special place in the One Health concept, where the health of the donor pigs is important for the health of the transplant recipient [90]. The sensitive methods developed to detect porcine viruses including HEV and the elimination programs developed for the donor pigs for xenotransplantation can also be applied to eliminate HEV from pigs produced for food. This will reduce the number of HEV infection in humans. The way of elimination of HEV in pig colonies generated for xenotransplantation can teach how to stop this emerging infection worldwide.

## 7. Conclusions

In order to prevent transmission of HEV from animals to humans and to stop the transmission from human to human by blood transfusion, effective counter-measures have to be undertaken, including treatment of contaminated food and water as well as elimination of HEV from pigs and other animals that are used for food production. This will be an enormous effort, but the results have the potential to guarantee an economic benefit and an improvement in public health.
